# Rescue Surgery 19 Years after Composite Root and Hemiarch Replacement

**DOI:** 10.1155/2013/619282

**Published:** 2013-04-10

**Authors:** Konstantin von Aspern, Joerg Seeburger, Christian D. Etz, Matthias Sauer, Lukas Lehmkuhl, Martin Misfeld, Friedrich W. Mohr

**Affiliations:** ^1^Department of Cardiovascular Surgery, Heart Center Leipzig, Struempellstraße 39, Saxony, 04289 Leipzig, Germany; ^2^Department of Diagnostic and Interventional Radiology, Heart Center Leipzig, Struempellstraße 39, Saxony, 04289 Leipzig, Germany

## Abstract

A 59-year-old male patient with Marfan's syndrome was referred to our clinic due to acute chest pain. His medical history contains complex surgery for type A aortic dissection 19 years ago including composite root replacement using a mechanical aortic valve. Immediate computed tomography indicated perforation at the distal ascending aortic anastomosis plus complete avulsion of both coronary ostia. The patient underwent successful rescue surgery with ascending aortic and arch replacement using a modified Cabrol technique.

## 1. Introduction

Late graft failure after ascending aortic replacement with a Dacron tube is extremely rare [1]. Herein we report a case of late prosthesis failure 19 years after a classic Bentall procedure with hemiarch and thoracoabdominal aortic replacement. The operative approach for rescue surgery is discussed.

## 2. Case Presentation

A 59-year-old male patient with Marfan's syndrome was referred for emergency surgery due to new onset of severe chest pain and dyspnea. Nineteen years ago the patient underwent a two-staged procedure including a composite graft replacement of the aortic valve, the ascending aorta (classic Bentall procedure), and hemiarch replacement in a first stage. At that time the aortic valve and ascending aorta were implanted with the aneurysmal sac wrapping the prosthesis. In a second stage eight months later the thoracoabdominal aorta up to the coeliac trunc was replaced.

**Figure 1:**
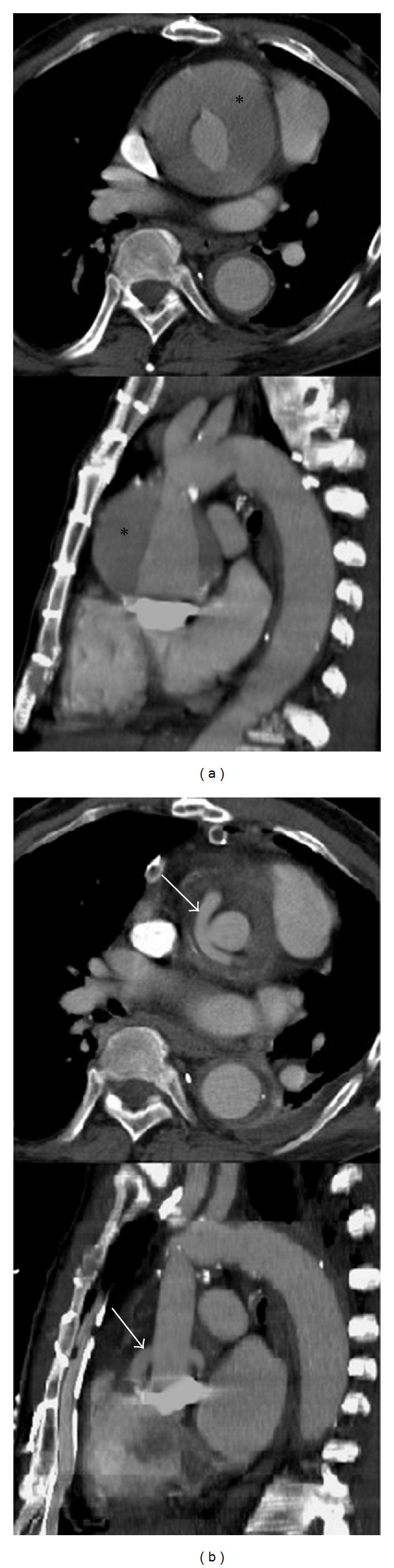
((a): top and bottom) Preoperative contrast enhanced CT scan showing the ascending aortic prosthesis, the mechanical aortic valve, and the surrounding hemorrhage within the former aneurysmal sac (black asterisk). ((b): top and bottom) Postoperative contrast enhanced CT scan showing the Cabrol interposition graft (white arrows).

On admission contrast enhanced computed tomography (CT) revealed perforation at the suture line of the distal ascending aortic prosthesis and avulsion of both coronary ostia (Figure 1(a)). Additionally, formation of a pseudoaneurysm with dissection of the right subclavian artery was present.

The patient underwent a median resternotomy. Peripheral femoral cannulation was used for cardiopulmonary bypass (CPB). The brachiocephalic vein (anonyma) was ligated and severed in order to sufficiently expose the dissected aneurysm of the right subclavian artery for later reconstruction. After complete resection of the former aortic aneurysm, the conduit was exposed and a suture dehiscence at the distal aortic anastomosis plus complete avulsion of both coronaries from the proximal prosthesis was verified. 

Cannulation of both carotid arteries for bilateral selective cerebral perfusion (SCP) was performed after exposure under direct vision. In order to prevent back flow/steal, the subclavian artery was blocked via a balloon-tipped catheter. SCP blood flow was 900 mL per minute at 25°C. Cerebral oxygenation was monitored by means of near-infrared spectroscopy using two optodes positioned bilaterally over both frontal lobes. Cardioplegia (Bretschneider HTK solution; Köhler Chemie, Alsbach-Hähnlein, Germany) was administered into both coronaries. A 22 mm Dacron prosthesis with four sidearms (Hemashield Platinum, Flagstaff, Arizona, USA) was selected for ascending aortic/arch replacement. The prosthesis was anastomosed end-to-end to the old descending aortic prosthesis. The left subclavian artery was reinserted into the prosthesis and perfusion via the femoro-femoral bypass was resumed. Subsequently the left and right carotid arteries were inserted into the prosthesis via the sidearms after resection of the innominate artery using short circulatory arrest for five and seven minutes, respectively. The remaining sidearm was directly connected to the right axillary arteries. The old aortic valve conduit and the new aortic prosthesis were reconnected end-to-end. Finally, the left and right coronary artery were reinserted into the composite graft using an eight mm Dacron graft applying the Cabrol technique [2] with one side-to-side anastomosis (Figure 1(b)). Total cardiac ischemic time was 120 minutes. Lower body temperature was 26°C. Weaning from CPB was uneventful.

Postoperative course was impaired due to stroke immediately after surgery with signs of right-sided hemiplegia. CT revealed hemorrhage of the basal ganglia. Despite that, the patient regained adequate consciousness and was transferred to a specialized neurology rehabilitation center six days after surgery. Predischarge CT of the aorta showed a regular postoperative result (Figure 1(b)).

## 3. Discussion

In case of dissection Marfan disease regularly requires aggressive surgery of the thoracic and/or abdominal aorta. Aneurysm formation and associated risk of perforation or rupture are lethal complications [3]. Vessel prostheses such as polyethylene terephthalate (PET or Dacron) or polytetrafluoroethylene (PTFE) are widely used with excellent long-term durability. Despite graft expansion up to 17% in diameter within the first seven postoperative days, which then slows down to less than 1% per year, graft failure such as leakage and/or perforation is rare [1].

Two decades ago, the patient had undergone a classic Bentall procedure with reincorporation of the coronaries by suturing the adjacent aortic wall to the perimeter of holes in the prosthesis. The wall of the aneurysm had been closed over the prosthesis, as originally described by Bentall and de Bono in 1968 [4]. This technique was thought to provide physical stabilization of the anastomoses in case of pseudoaneurysm formation and creates an immobile space between the prosthesis and the native aortic wall to prevent potential leakage [5]. The potential advantage of averting free perforation in case of suture line failure is traded in for the risk of coronary stretch, as in this case. Assuming that graft failure originated from the suture line of the distal ascending aortic anastomosis, resulting in leakage into the former aneurysm, the increasing shear stress generated by the progressive pressurization of the former aneurysm sac might have caused the coronary ostia to tear away from the prosthesis. Although this disadvantage of the classic Bentall procedure is widely known, graft failure almost two decades after the initial surgery has to the best of our knowledge not been previously reported. Late complications in Marfan patients occur on average 8 years after the initial surgery [6, 7]. These complications include formation of aneurysm/pseudoaneurysm at the proximal/distal aortic anastomosis and at the site of coronary reimplantation depending on the utilized technique.

Today the modified Bentall procedure implementing a “button” technique for coronary reimplantation is considered the procedure of choice for aortic root replacement [3].

In general, structural damage to the integrity of the graft due to instrument handling during the initial surgery might be a source for late graft failure. Correct cutting of the material with a wire cautery device to seal the fabric edges instead of blunt cutting might further reduce this risk [8]. The influence of arterial hypertension on late complications after root surgery is not evaluated completely. According to retrospective clinical reports, however, our patient had an adequate antihypertensive regime with oral medication. Nevertheless, undocumented recurrent acute blood pressure peaks might also contribute to late graft failure. Therefore we believe that a strict antihypertensive regime is mandatory.

The selected approach for redo-surgery in this case was to implement a Cabrol coronary interposition graft [2]. Due to the necessity of proximal coronary resection and subsequently shortening of both coronary arteries as a result of the traumatic tear-off, an interposition graft was used to bridge the distance, thereby avoiding excessive traction and coronary kinking. Experience regarding longevity of an isolated Cabrol interposition graft (without an additional Cabrol fistula) after redo-surgery is sparse. A nonnegligible incidence of complications after this procedure due to ostial stenosis of the single lumen with or without graft torsion and kinking has been reported [9]. Accordingly, anastomoses for the left and right coronary arteries by means of two separate short interposition grafts might have also been an option. However, surgeons choice deemed a single graft best suited in this particular case.

## 4. Conclusions

In summary it should be noted that late graft failure is a very rare but serious complication and can occur as late as two decades after the initial surgery. Therefore, regular check-ups are recommended. Utilization of suture-graft inclusion in the initial surgery in combination with the modified Bentall procedure for composite aortic root replacement can serve as backup in case of graft failure, especially in the presence of connective tissue disease. If such a complication does occur, the Cabrol technique with a coronary interposition graft is a feasible method as first-line therapy for redo-surgery.
